# Transcriptional dynamics of granulocytes in direct response to incubation with SARS‐CoV‐2

**DOI:** 10.1002/2211-5463.13500

**Published:** 2022-11-28

**Authors:** Daigo Nakazawa, Yohei Takeda, Masatoshi Kanda, Utano Tomaru, Haruko Ogawa, Takashi Kudo, Satoka Shiratori‐Aso, Kanako Watanabe‐Kusunoki, Yusho Ueda, Atsuko Miyoshi, Fumihiko Hattanda, Saori Nishio, Ryo Uozumi, Akihiro Ishizu, Tatsuya Atsumi

**Affiliations:** ^1^ Department of Rheumatology, Endocrinology, and Nephrology, Faculty of Medicine and Graduate School of Medicine Hokkaido University Sapporo Japan; ^2^ Research Center for Global Agromedicine Obihiro University of Agriculture and Veterinary Medicine Japan; ^3^ Department of Veterinary Medicine Obihiro University of Agriculture and Veterinary Medicine Japan; ^4^ Department of Rheumatology and Clinical Immunology Sapporo Medical University Japan; ^5^ Department of Pathology, Faculty of Medicine and Graduate School of Medicine Hokkaido University Sapporo Japan; ^6^ Division of Laboratory and Transfusion Medicine Hokkaido University Hospital Sapporo Japan; ^7^ Department of Medical Laboratory Science, Faculty of Health Sciences Hokkaido University Sapporo Japan

**Keywords:** granulocytes, low‐density granulocytes, neutrophil extracellular traps, SARS‐CoV‐2, severe COVID‐19

## Abstract

Severe coronavirus disease 2019 (COVID‐19) is characterized by acute respiratory distress syndrome and multiple organ dysfunction, in which the host immune response plays a pivotal role. Excessive neutrophil activation and subsequent superfluity of neutrophil extracellular traps (NETs) can lead to tissue damage, and several studies have shown the involvement of neutrophils in severe COVID‐19. However, the detailed responses of each neutrophil subset to SARS‐CoV‐2 infection has not been fully described. To explore this issue, we incubated normal‐density granulocytes (NDGs) and low‐density granulocytes (LDGs) with different viral titers of SARS‐CoV‐2. NDGs form NETs with chromatin fibers in response to SARS‐CoV‐2, whereas LDGs incubated with SARS‐CoV‐2 display a distinct morphology with condensed nuclei and moderate transcriptional changes. Based on these transcriptional changes, we suggest that AGO2 possibly plays a role in LDG regulation in response to SARS‐CoV‐2.

## Graphical abstract

In coronavirus disease 2019, excessive neutrophil activation leads to tissue damage. In this study, we observed morphological and transcriptional changes of low‐density granulocytes (LDGs) induced by incubation with SARS‐CoV‐2 *ex vivo*. In particular, the AGO2 gene, which is involved in the biogenesis and function of miRNAs, was detected as a key regulator. AGO2 possibly plays a role in LDG regulation in response to SARS‐CoV‐2.

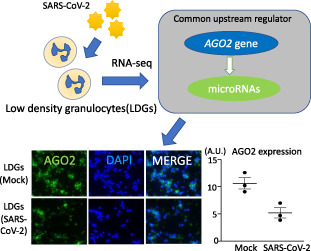

**Abbreviations**
ACE2: angiotensin‐converting enzyme 2
AGO2: argonaute RISC catalytic component 2
ARDS: acute respiratory distress syndrome
COVID19: coronavirus disease 2019
DAMPs: damage‐associated molecular patterns
FDR: false discovery rate
GO: Gene Ontology
ISGs: interferon‐stimulated genes
LDGs: low‐density granulocytes
MOD: multiple organ dysfunction
NDGs: normal‐density granulocytes
NETs: neutrophil extracellular traps
PBMC: peripheral blood mononuclear cell
TUNEL: TdT‐mediated dUTP‐biotin nick end labeling

Coronavirus disease 2019 (COVID‐19) caused by severe acute respiratory syndrome coronavirus 2 (SARS‐CoV‐2) has emerged as a pandemic. Patients with severe COVID‐19 usually develop acute respiratory distress syndrome (ARDS) and multiple organ dysfunction (MOD), resulting in high mortality rate [1]. Several studies have revealed that severe disease is associated with vascular thrombosis due to the COVID‐19 coagulopathy presumably caused by excessive immune responses [2]. In mild cases, SARS‐CoV‐2 affects only T‐ and B‐cell activation, but an elevated neutrophil count is observed in patients with severe COVID‐19 [3]. Single‐cell RNA sequencing from the peripheral blood sample of patients with severe disease revealed the presence of dysregulated myeloid cell compartments with altered myelopoiesis and neutrophil maturation leading to the development of COVID‐19 [4]. Recent studies have suggested that some subtypes of neutrophils play a role in the pathogenesis of various diseases including cancer and autoimmune diseases. Normal‐density granulocytes (NDGs) and low‐density granulocytes (LDGs) are found in sediment on the red blood cells and in low‐density fraction including mononuclear cells, respectively. Under unsteady‐state conditions, NDGs and LDGs exhibit specific immunological response; including phagocytosis, degranulation, neutrophil extracellular trap (NET) formation, and expression of immune‐related genes [5, 6]. NETs were detected in the circulation and organs of patients with severe COVID‐19 [7]. They are composed of decondensed chromatin packed with antiviral proteins including histone and myeloperoxidase, which could potentially prevent virus spread [8]. Additionally, they influence the coagulant system by developing a scaffold thrombus to enclose the virus effectively, a process named as immunothrombosis [9]. However, excessive NETs and immunothrombosis can cause tissue damage, particularly in patients with severe COVID‐19. Furthermore, LDGs in such patients not only form NETs but also display transcriptional changes linked to neutrophil recruitment and activation [10]. Although *in vivo* granulocytes of such patients have been characterized, the behavior of SARS‐CoV‐2 on neutrophils remains unclear; that is, detailed responses to SARS‐CoV‐2 infections in each neutrophil subset have not been fully established. It is supposed that SARS‐CoV‐2 mechanistically affects neutrophils via the entry of angiotensin‐converting enzyme 2 (ACE2) [11] and CD147 [12]. In the present study, we aimed to explore a novel approach by evaluating the molecular mechanisms and transcriptional profiles of NDGs and LDGs in response to SARS‐CoV‐2 *ex vivo*.

## Materials and methods

### Neutrophil isolation

Blood samples from three healthy donors were collected for the experiments. NDGs were isolated from these blood samples by density gradient centrifugation method using PolymorphPrep® (Axis‐Shield, Dundee, UK). Neutrophils were suspended in RPMI 1640 (Merck, Darmstadt, Germany) and seeded into sterile plates for incubation in 5% CO_2_ at 37 °C. To purify LDGs, peripheral blood mononuclear cells (PBMCs) were isolated from whole blood samples of healthy donors using Ficoll‐Paque® (Cytiva, Marlborough, MA, USA) according to the manufacturer's instructions. LDGs were then isolated from PBMCs using the EasySep Human Neutrophil Negative Selection Kit® (STEMCELL, Vancouver, BC, Canada), as described previously [13]. The purity of isolated NDGs and LDGs estimated by Gimusa staining was 95–97% and 90–95%, respectively. Experiments using human materials were permitted by the Hokkaido University Hospital Clinical Research Committee (Approval number; 020‐0283). Written informed consent was obtained from the participants. All procedures were carried out in accordance with the ethical code of the Declaration of Helsinki.

### Preparation of SARS‐CoV‐2 solution

JPN/TY/WK‐521 strain of SARS‐CoV‐2 was provided by the National Institute of Infectious Diseases (Tokyo, Japan). VeroE6/TMPRSS2 cell was obtained from the Japanese Collection of Research Bioresources (Osaka, Japan, Cell number: JCRB1819). SARS‐CoV‐2 was inoculated to the VeroE6/TMPRSS2 cells cultured in Dulbecco's modified Eagle's medium (Nissui Pharmaceutica, Tokyo, Japan) supplemented with 1% (v/v) fetal bovine serum, 2 mm l‐glutamine (Fujifilm Wako Pure Chemical, Osaka, Japan), 100 μg·mL^−1^ kanamycin (Meiji Seika Pharma, Tokyo, Japan), and 2 μg·mL^−1^ amphotericin B (Bristol‐Myers Squibb, New York, NY, USA). After 3 days, the cell culture supernatant was collected and used as a SARS‐CoV‐2 solution. Based on the degree of cytopathic effect observed on VeroE6/TMPRSS2 cells, the viral titer of SARS‐CoV‐2 solution was determined to be 7.0 log_10_ 50% tissue culture infective dose (TCID_50_)·mL^−1^.

### Co‐culture of neutrophils with SARS‐CoV‐2

Normal‐density granulocytes (1 × 10^6^ cells·mL^−1^) and LDGs (1 × 10^5^ cells·mL^−1^) isolated in previous step were incubated in RPMI 1640 containing 2% (v/v) fetal bovine serum. For microscopic analysis, a total of 2 × 10^5^ NDGs and 2 × 10^4^ LDGs were incubated with SARS‐CoV‐2 (−0.7 to 5.3 log_10_ TCID_50_/200 μL) for 4 h at 37 °C. For RNA isolation, a total of 3 × 10^6^ NDGs and 3 × 10^5^ LDGs were incubated with SARS‐CoV‐2 (0.5–6.5 log_10_ TCID_50_/3 mL) for 4 h at 37 °C. For the mock, the cell culture supernatant from VeroE6/TMPRSS2 cells without SARS‐CoV‐2 infection was added to NDGs and LDGs. The experiments of LDGs were conducted within available cell volume.

### Immunostaining

The phase‐contrast microscopic images were recorded to observe the morphological changes in neutrophils in response to SARS‐CoV‐2 inoculation. Immunostaining for neutrophils were performed using anti‐citrullinated histone (CitH3) antibody (Abcam, Cambridge, UK), anti‐MPO antibody (Abcam), anti‐BPI antibody (Santa Cruz Biotechnology, Santa Cruz, CA, USA), and anti‐AGO2 antibody (Fujifilm Wako Pure Chemical) after fixation with 4% paraformaldehyde. Neutrophil death was evaluated by TdT‐mediated dUTP‐biotin nick end labeling (TUNEL) assay. The positive area of TUNEL staining and immunostaining was quantified using the imagej software (National Institutes of Health, Bethesda, MD, USA). The data represent mean ± SEM values of three independent experiments.

### RNA isolation and RNA sequencing

Total mRNA was isolated from NDGs and LDGs using an Isogen II Kit (Nippon Gene, Tokyo, Japan) according to the manufacturer's instructions. The quality of extracted RNA was evaluated using a Bioanalyzer 2100, and the total RNA concentration was determined using Qubit RNA assay kits (Thermo Fisher Scientific, Waltham, MA, USA). The rRNAs were depleted using the NEBNext rRNA Depletion Kit (New England Biolabs, Ipswich, MA, USA; #E6310S/L/X) and sequencing libraries were prepared using NEBNext Ultra II Directional RNA Library Prep Kit for Illumina (San Diego, CA, USA) (New England Biolabs; #E7760S/L), according to the manufacturer's instructions. Sequencing was performed on the NovaSeq 6000 (Illumina) platform in a 2 × 150 bp paired‐end configuration with over 20 million reads per samples. Raw sequencing reads with low‐quality and adapter sequences were removed using cutadapt v 2.1 (DOI: https://doi.org/10.14806/ej.17.1.200). The trimmed reads were mapped to hg38 (Ensembl version 93) and quantified using star aligner v 2.5.1 [14]. Differentially expressed genes (DEGs) were identified based on the differences in expression levels (¦log_2_ fold‐change¦ ≥ 1 and adjusted *P*‐value < 0.05) between samples after removing genes with zero read count using deseq2 v 1.28.1 [15] or edger v 3.30.3 [16]. Gene Ontology (GO) analysis was performed using the gprofiler2 r package v 0.2.1 [17]. The raw data were submitted to DDBJ (Accession number: DRA014601, BioProject: PRJDB13935).

### Ingenuity pathway analysis

The changes in gene expression level were considered significant if their fold change value was > 1.5× in either direction, or the false discovery rate (FDR)‐corrected statistical significance was < 0.05. To explore the biological processes by transcriptome analysis, upstream pathway analysis and canonical pathway analysis of DEGs were performed using ingenuity pathway analysis (ipa ®) v 51963813 (Qiagen, Redwood City, CA, USA).

### Statistical analysis

Statistical analyses were performed using r v 3.6.1 (R Foundation for Statistical Computing, Vienna, Austria, https://www.R‐project.org/) or prism 6 (GraphPad Software, San Diego, CA, USA). The data were analyzed using one‐way ANOVA with *post hoc* Tukey, one‐way ANOVA followed by Dunnett's multiple comparisons test or Student's *t*‐test to calculate statistical significance, with results being considered statistically significant at *P* < 0.05. The Benjamini–Hochberg method was used to adjust the *P*‐value for multiple hypothesis testing. All results were presented as the mean ± SEM.

## Results

### SARS‐CoV‐2 influences NDGs to induce neutrophil death with NETs release *ex vivo*

**Fig. 1:**
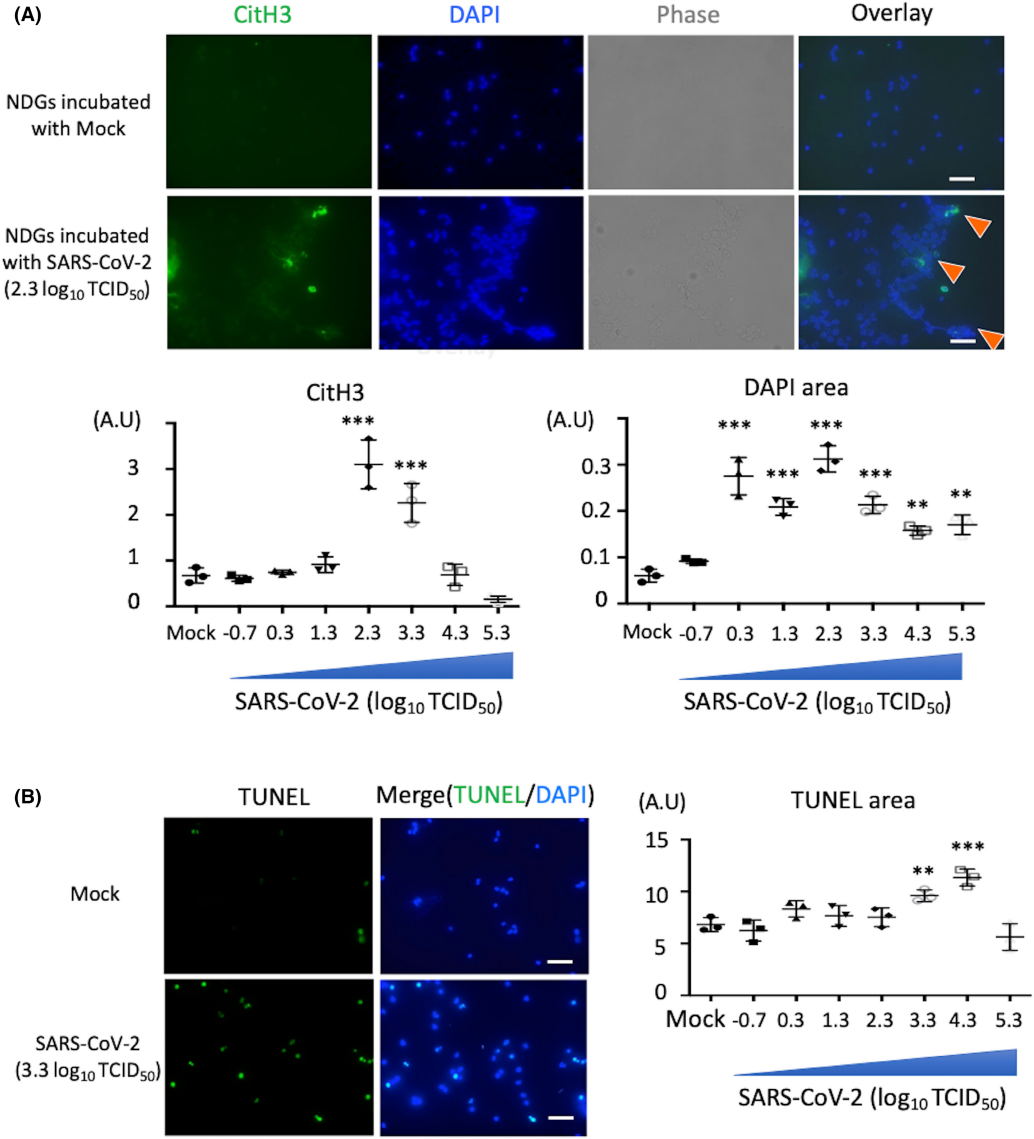
SARS‐CoV‐2 influences NDGs to induce NETs formation. (A) NDGs were isolated from healthy volunteers by polymorphprep and incubated with mock or SARS‐CoV‐2 (viral titer: 2.3 log_10_ TCID_50_) for 4 h (*n* = 3/group). Upper figures show the representative images of NET release with citrullinated histone H3 (CitH3). Cells were stained for nuclei (4′,6‐diamidino‐2‐phenylindole: DAPI, blue) and CitH3 (NETs marker, green). Arrowheads show NETs formation with CitH3. Lower graphs show dot plots for the CitH3 or DAPI positive area in NDGs incubated with SARS‐CoV‐2 in a viral titer dependent manner (−0.7 to 5.3 log_10_ TCID_50_). (B) The representative images (left) and positive area (right) of TUNEL assay in NDGs incubated with SARS‐CoV‐2 (*n* = 3/group). Data are presented as mean ± SEM. One‐way ANOVA with Dunnett's multiple comparisons test (vs. mock) was performed for statistical analyses, and significance was defined as **P < 0.01, ***P < 0.001. Scale bar; 50 μm.

Various stimuli, including viruses, bacteria, damage‐associated molecular patterns (DAMPs), autoantibodies, and inflammatory cytokines, influence neutrophils to induce NET formation [18]. Recent studies revealed that SARS‐CoV‐2 also triggers NET formation *ex vivo* [11], and alters the neutrophil heterogeneity *in vivo* as observed in the peripheral blood samples of patients with COVID‐19 [19]. To validate the precise neutrophil response against SARS‐CoV‐2, two distinct neutrophil subsets derived from healthy donors were incubated with different viral titers of SARS‐CoV‐2. The NDGs cultured with SARS‐CoV‐2 for 4 h resulted in NET‐DNA release with histone citrullination, a NET marker. The level of citrullinated histones and swollen nuclei was highest at 2.3 log_10_ TCID_50_ of SARS‐CoV‐2 (Fig. 1A). Since phorbol myristate acetate‐induced NETosis reportedly differs from apoptosis, which is characterized by condensed nuclei and TUNEL positivity [20], we performed TUNEL assay to identify the population of dead neutrophils in response to SARS‐CoV‐2. Results show TUNEL‐positive NDGs in the presence of a high viral titer of SARS‐CoV‐2 (Fig. 1B). These findings indicate that neutrophils with histone citrullination and TUNEL‐positivity exist in combined state in response to SARS‐CoV‐2.

### SARS‐CoV‐2 affects LDGs to induce histone citrullination with morphological alteration

**Fig. 2:**
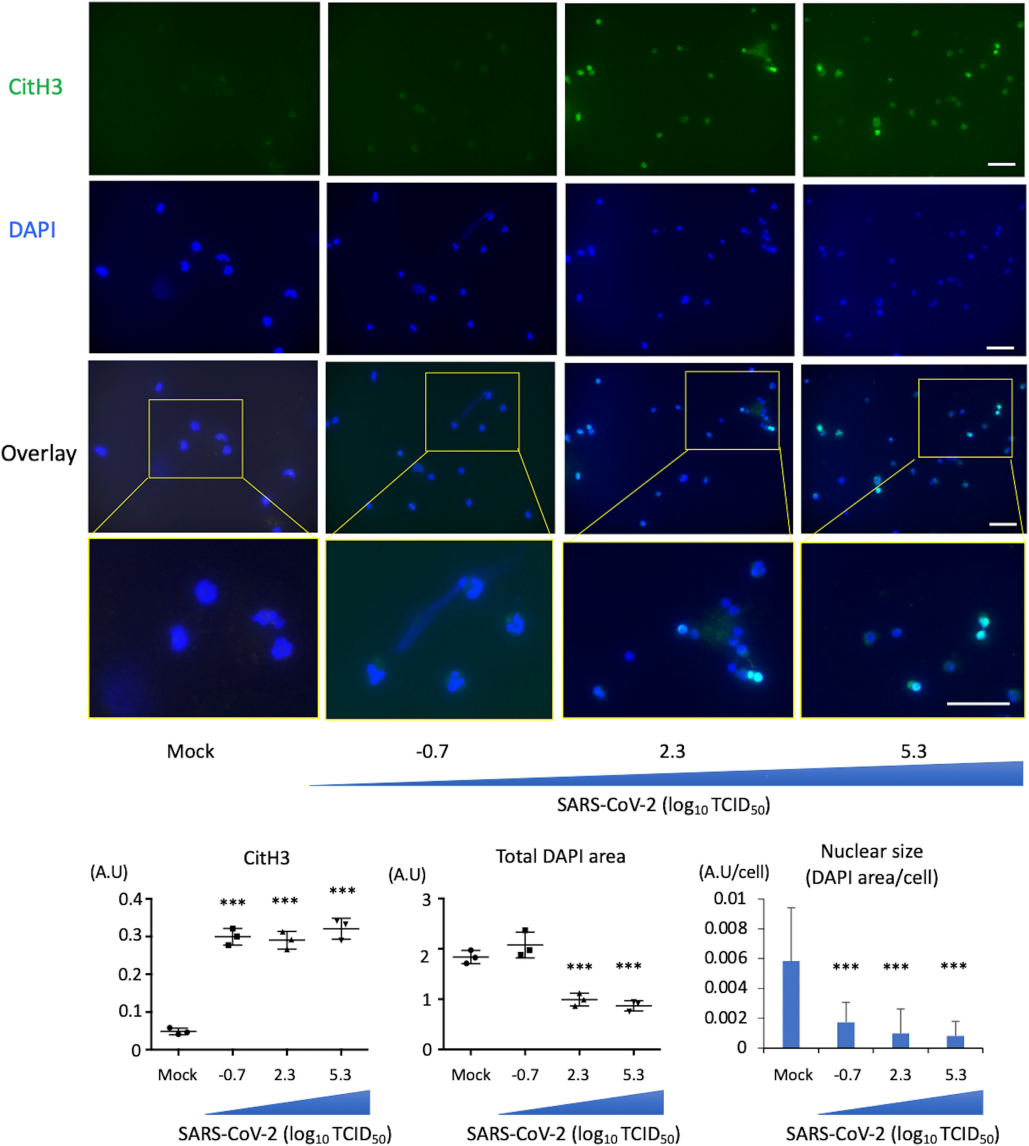
SARS‐CoV‐2 influences LDGs to alter the morphology with histone citrullination. (A) PBMCs were isolated by ficoll from healthy donors and the LDGs were separated using a neutrophil isolation kit from PBMCs. LDGs were incubated with mock or SARS‐CoV‐2 (−0.7 to 5.3 log_10_ TCID_50_) for 4 h (*n* = 3/group). Upper figures show representative images in each group (4′,6‐diamidino‐2‐phenylindole: DAPI, blue; citrullinated histone h3: CitH3, green). Lower graphs show CitH3 area, total DAPI area, and the nuclear size (total DAPI area adjusted by cell numbers) of LDGs incubated with various viral titers of SARS‐CoV‐2 (−0.7, 2.3, and 5.3 log_10_ TCID_50_). Data are presented as mean ± SEM. One‐way ANOVA with Dunnett's multiple comparisons test (vs. mock) was performed for statistical analyses, and significance was defined as ***P < 0.001. Scale bar; 50 μm.

It has been suggested that LDGs, the circulating neutrophil subsets, are involved in pathogenesis of autoimmune diseases, cancer, and infection by inducing inflammation. According to the recent reports, the number of LDGs increased in patients with COVID‐19, and circulating LDGs exhibited active state and altered heterogeneous dynamics [4]. To evaluate the direct effect of SARS‐CoV‐2 on LDGs *ex vivo*, LDGs from healthy donors were cultured with SARS‐CoV‐2 as mentioned in Co‐culture of neutrophils with SARS‐CoV‐2 section. As a result, histone citrullination increased with virus titer. However, in contrast to NDGs, the nuclei of LDGs were contracted by incubation with viruses (Fig. 2).

### Transcriptional profile of NDGs and LDGs incubated with SARS‐CoV‐2

**Fig. 3:**
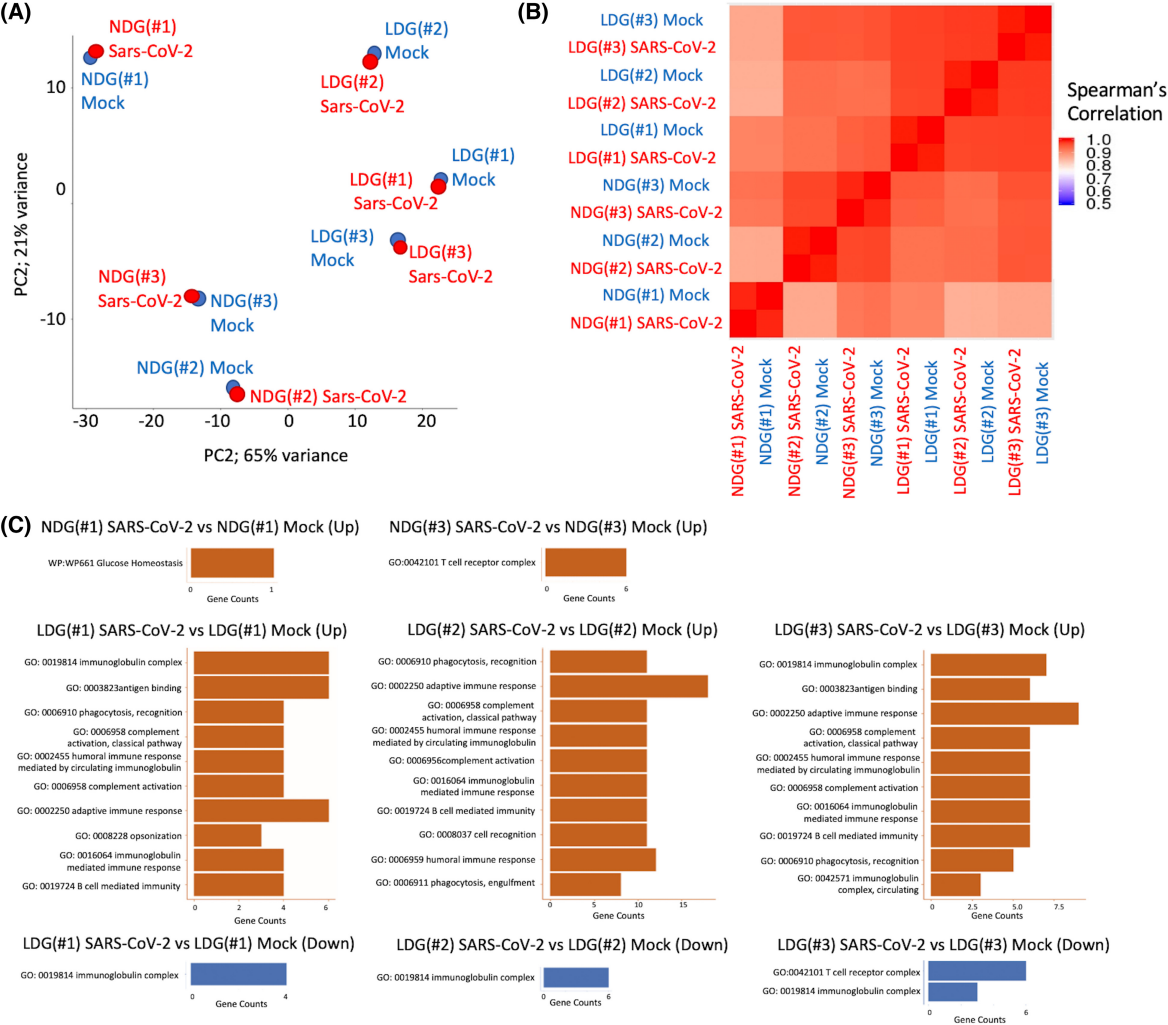
The transcriptional profiles in NDGs and LDGs incubated with SARS‐CoV‐2 *ex vivo* experiments. (A, B) Principal component analysis (A) and Heatmap of Spearman's correlation coefficients (B) of three healthy donor (#1–#3)‐derived NDGs and LDGs incubated with mock (blue) or SARS‐CoV‐2 (red). (C) The GO biological process terms which corresponded to coding gene function of upregulated and downregulated in RNA sequencing data of neutrophils incubated with SARS‐CoV‐2 compared to neutrophils incubated with mock.

**Fig. 4:**
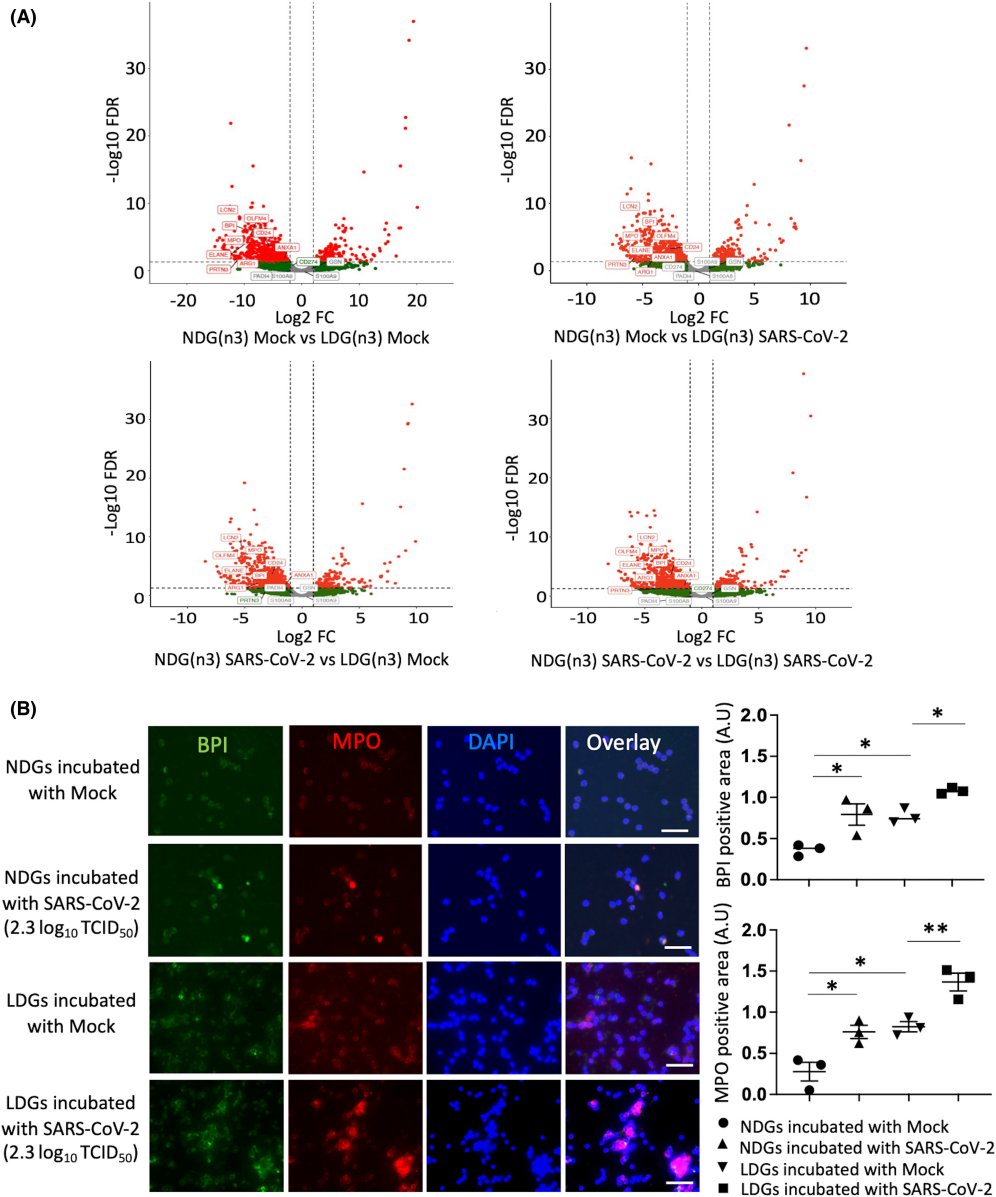
(A) Volcano plots of gene expression between NDGs and LDGs incubated with mock or SARS‐CoV‐2 were visualized. Protein‐coding genes that pass the thresholds for FDR and log fold change (FC) (FDR <0.05 and ¦log_2_ FC¦ > 1) were plotted in red. The characteristic genes for LDGs were mapped. (B) The immunostaining of BPI and MPO in NDGs and LDGs incubated with mock or SARS‐CoV‐2 (2.3 log_10_ TCID_50_) (for 4 h incubation, *n* = 3/group). Green: BPI, red: MPO, blue: DAPI staining. All data in the graph are presented as mean ± SEM. Statistical significance was assessed using one‐way ANOVA with *post hoc* Tukey and significance was defined as *P < 0.05, **P < 0.01. Scale bar; 50 μm.

**Fig. 5:**
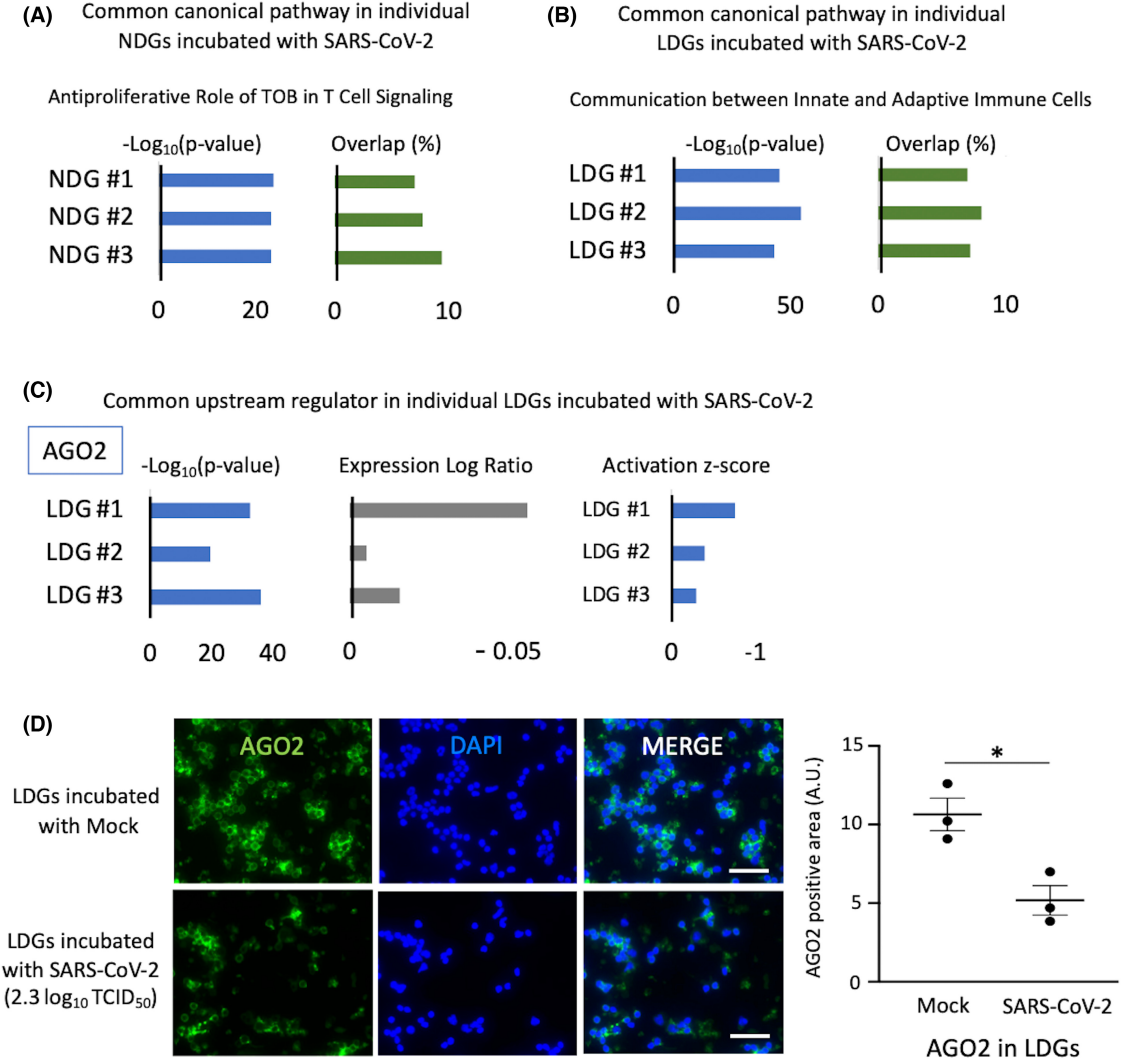
(A, B) Canonical pathway in RNA sequencing data of individual neutrophils incubated with SARS‐CoV‐2 compared to neutrophils incubated with mock (A: NDGs, B: LDGs). The shown data was identified as a common pathway among granulocytes of individuals. (C) The potential upstream regulator commonly identified in individual LDGs incubated with SARS‐CoV‐2. (D) The immunostaining of AGO2 in LDGs incubated with mock or SARS‐CoV‐2 (2.3 log_10_ TCID_50_) (for 4 h incubation, *n* = 3/group). Green: AGO2, Blue: DAPI staining. Data in graph are presented as mean ± SEM. Statistical significance was assessed using Student's *t*‐test and significance was defined as *P < 0.05. Scale bar; 50 μm.

To investigate the transcriptional mechanisms underlying the neutrophil response to SARS‐CoV‐2, NDGs, and LDGs collected from independent healthy donors were incubated with SARS‐CoV‐2 and mock, and transcriptome profiling was performed by RNA sequencing. Principal component analysis and Spearman's correlation analysis revealed that the transcriptomic changes in NDGs or LDGs by incubation with SARS‐CoV‐2 were less than those of the cell type or individual differences (Fig. 3A,B). To evaluate the transcriptomic changes induced by SARS‐CoV‐2 precisely, DEGs were analyzed per each donor in each cell type (NDGs or LDGs). In NDGs incubated with SARS‐CoV‐2, there were few DEGs and GO analysis showed almost no enrichment in GO terms; however, in LDGs incubated with SARS‐CoV‐2, there were stronger transcriptomic changes than those of NDGs. GO analysis in the LDGs revealed their association with immunological responses, including innate and adaptive immune responses, phagocytosis, and complement activation (Fig. 3C). The transcriptomic profiles comparing NDGs and LDGs were similar substantially regardless of the presence of SARS‐CoV‐2 (Fig. 4A and Tables S1–S4). A large number of protein‐coding genes were differentially regulated between NDGs and LDGs and characteristic genes of LDGs including bactericidal/permeability‐increasing protein (*BPI*), myeloperoxidase (*MPO*), *PRTN3*, and *ELANE*(4) were upregulated in LDGs regardless of the presence of SARS‐CoV‐2. The presence of SARS‐CoV‐2 did not impact on the gene expression levels of *BPI* and *MPO* in both NDGs and LDGs (Fig. 4A). In the analysis of protein expression, immunostaining showed higher intensity of BPI and MPO in LDGs. Interestingly, the SARS‐CoV‐2 enhanced the positivity of BPI and MPO in both NDGs and LDGs (Fig. 4B). Canonical pathway analysis using IPA showed the involvement of ‘Antiproliferative Role of TOB in T‐Cell Signaling’ in NDGs incubated with SARS‐CoV‐2 and ‘Communication between Innate and Adaptive Immune Cells’ in LDGs incubated with SARS‐CoV‐2 (Fig. 5A,B, and Fig. S1). Furthermore, argonaute RISC catalytic component 2 (*AGO2*) was detected as a common regulator by upstream analysis of LDGs incubated with SARS‐CoV‐2. The expression of *AGO2* in LDGs was higher than that in NDGs (Fig. S2A), which was validated by AGO2 immunostaining (Fig. S2B). In addition, the expression of *AGO2* transcript tended to be down‐regulated in LDGs incubated with SARS‐CoV‐2 (Fig. 5C). In immunostaining, the positivity of AGO2 protein in LDGs was reduced by incubation with SARS‐CoV‐2 (Fig. 5D). These findings indicated that AGO2 in LDGs incubated with SARS‐CoV‐2 might play a role in the physiology of LDGs.

## Discussion

*In vivo* neutrophils and NETs in patients with severe COVID‐19 have previously been linked to the pathogenesis of severe COVID‐19; however, the molecular and transcriptional mechanisms of neutrophils response to SARS‐CoV‐2 have been poorly understood. Our data showed that *ex vivo* NDGs and LDGs react morphologically and genetically with SARS‐CoV‐2 and display different phenotypes. The NDGs showed NETosis via histone‐citrullination, with minimal transcriptional changes. Conversely, LDGs displayed morphological changes with condensed nucleus with minimal transcriptional changes for immunological responses. *AGO2*, which is involved in the biogenesis and function of miRNAs [21], was detected as a key regulator of LDGs in response to SARS‐CoV‐2. Mortality due to COVID‐19 has been strongly associated with diffused alveolar damage and MOD [22]. The pathogenesis of these complications in response to SARS‐CoV‐2 is supposed to be caused by the interaction between pulmonary alveolar epithelial cells, immune cells, endothelial cells, and aberrant cytokines, leading to vascular dysfunction and coagulation [23]. In particular, neutrophils have drawn significant attention as triggers for this complex mechanism. An increased neutrophil/lymphocyte ratio was reported in the peripheral blood of patients with severe COVID‐19 [24]. The pathological findings from autopsies demonstrated NET formation in damaged organ vessels, including pulmonary capillaries, which are co‐localized with fibrin deposition occasionally [7, 25]. Neutrophils act as the first defenders against microbes by releasing radical oxygen species, initiating phagocytosis, and forming NETs [26]. NETs release cytotoxic chromatin fibers associated with antibacterial proteins into the extracellular space to kill the microbes effectively [27]. During viral infection, virus induces NETs formation and the components of formed NETs can inactivate and mobilize the virus by their antiviral properties. Moreover, NETs interact with platelets and coagulant system to produce scaffold thrombi, a process named immunothrombosis, which enclose the microbes efficiently. However, other triggers, including DAMPs, crystals, and cytokines, which are associated with various diseases, also induce NET formation and immunothrombosis, causing vascular damage through sterile inflammation [18, 28]. Furthermore, the significance of neutrophil diversity under steady and crisis conditions is well known, which also influences the characteristics of NET formation. In anti‐neutrophil cytoplasmic antibody‐associated vasculitis, NDGs tend to form cell lytic NETs with extracellular genomic DNA. Similarly, a subset of LDGs in systemic lupus erythematosus forms pro‐inflammatory NETs with enhanced expression of interferon‐stimulated genes (ISGs) [29, 30]. Thus, elucidating the neutrophil dynamics, including their diversity and molecular signaling pathways in COVID‐19, could explain the severe disease development. Schulte‐Schrepping et al. [4] explained the dynamics of neutrophil diversity, including their composition and activation state, in peripheral blood of severe COVID‐19 patients using single‐cell RNA sequencing analysis. In particular, increased LDGs contained eight transcriptionally distinct cell clusters, including pro‐neutrophils, pre‐neutrophils, and mature neutrophils, as defined by existing markers. Of these, one of mature neutrophil cluster and other pro‐neutrophil cluster in LDGs showed high expression of ISGs (*ISG15*, *IFITM1/3*, and *RSAD2*) and genes involved in NET formation (*MPO*, *ELANE*, and *PRTN3*), respectively. Cabrera et al. [31] also identified elevated LDGs with transcriptionally distinct subclusters in the PBMCs of patients with severe COVID‐19, suggesting a link with emergency myelopoiesis and neutrophil activation. Therefore, we examined the direct effect of SARS‐CoV‐2 on NDGs and LDGs *ex vivo*. We observed that SARS‐CoV‐2 affects NDGs to induce NETs with hypercitrullinated histones. This finding is supported by the previous report of Veras et al. [11], which showed that NET formation is dependent on post‐transcriptional regulation by peptidyl arginine deiminase (PADI) 4 via the entry of ACE2. Our study reported minimal transcriptional changes in NDGs incubated with SARS‐CoV‐2. Besides, LDGs incubated with SARS‐CoV‐2 displayed abnormal morphological changes with transcriptional changes, involving the immunological response. Although LDGs incubated with SARS‐CoV‐2 did not exhibit significant transcriptional changes in the key genes for NET formation like *MPO*, *ELANE*, *PRTN3*, and *PADI4*, the protein levels of neutrophil markers (BPI and MPO) were enhanced by SARS‐CoV‐2. Furthermore, *AGO2*, which plays a role in the biogenesis of miRNAs, was detected as a key regulator of LDGs in response to SARS‐CoV‐2. In addition, AGO2 confers resistance to viral replication and could be a therapeutic candidate for viral infection [32] and our data showed that the expression of *AGO2* was higher in the LDGs than the NDGs and the expression of AGO2 was down‐regulated by the incubation with SARS‐CoV‐2 in LDGs. From these facts, we assumed AGO2 might have protective effects on the viral load of SARS‐CoV‐2 in LDGs. The functional analysis of AGO2 in LDGs is needed for further studies. Meanwhile, in view of the findings from our *ex vivo* and previous *in vivo* data, it is assumed that peripheral LDGs respond to SARS‐CoV‐2 mainly via post‐transcriptional modifications, with minimal transcriptional changes, and *in vivo* peripheral LDGs with significant transcriptional changes are recruited mainly from the bone marrow in response to systemic conditions or are the consequence of activation caused by humoral factors and other immune cells in circulation. Patients with severe COVID‐19 have increased inflammatory immune cells, cytokine storms, hypercoagulability, and DAMPs derived from injured tissues in their circulation, which may affect the development and transcriptional regulation of bone marrow granulocytes. In addition to significant findings of our study, few limitations were also identified. First, NDGs and LDGs used in present study were derived from healthy donors with no risk factor for severe COVID‐19, which possibly resulted in minimal transcriptional changes. Second, our experimental model in which NDGs or LDGs alone are incubated with SARS‐CoV‐2 is not considering the effects of the other immune cells which have important interactive roles in driving inflammation *in vivo*. Third, the limited transcriptional changes might be caused by the differences, and finally neutrophils are terminally differentiated cells; therefore, proteomic analysis in the same direction is needed for future studies.

## Conflict of interest

The authors declare no conflict of interest.

## Author contributions

DN, YT and MK were involved in conceptualization, methodology, formal analysis, data curation and writing of the original draft. UT and AI were involved in conceptualization, methodology and data curation. HO, TK, SS‐A, KW‐K, YU, AM, FH, SN and RU were involved in conceptualization and methodology. TA was involved in conceptualization and data curation.

## Supporting information


**Fig. S1.** Canonical pathway analysis in individual granulocytes incubated with SARS‐CoV‐2 (Top 20).

**Fig. S2.** AGO2 in granulocytes.

**Table S1.** Highly Significant DEGs in NDGs incubated with Mock (n3) compared to LDGs incubated with Mock (n3) (Top50).
**Table S2.** Highly Significant DEGs in NDGs incubated with Mock (n3) compared to LDGs incubated with SARS‐CoV‐2 (n3) (Top50).
**Table S3.** Highly Significant DEGs in NDGs incubated with SARS‐CoV‐2 (n3) compared to LDGs incubated with Mock (n3) (Top50).
**Table S4.** Highly Significant DEGs in NDGs incubated with SARS‐CoV‐2 (n3) compared to LDGs incubated with SARS‐CoV‐2 (n3) (Top50).

## Data Availability

The data supporting the findings of this study are available from the corresponding author upon reasonable request.
