# Supplementary material for: Transcriptional dynamics of granulocytes in direct response to incubation with SARS‐CoV‐2
Source: FEBS Open Bio. 2022 Nov 28;13(1):60–71. doi: 10.1002/2211-5463.13500 (PMC9808587; doi:10.1002/2211-5463.13500)
Supplement: Supplementary file 3 — Table S1. Highly Significant DEGs in NDGs incubated with Mock (n3) compared to LDGs incubated with Mock (n3) (Top50). Table S2. Highly Significant DEGs in NDGs incubated with Mock (n3) compared to LDGs incubated with SARS‐CoV‐2 (n3) (Top50). Table S3. Highly Significant DEGs in NDGs incubated with SARS‐CoV‐2 (n3) compared to LDGs incubated with Mock (n3) (Top50). Table S4. Highly Significant DEGs in NDGs incubated with SARS‐CoV‐2 (n3) compared to LDGs incubated with SARS‐CoV‐2 (n3) (Top50). [file FEB4-13-60-s003.docx]

Supplemental Tables

Supplemental Table 1. Highly Significant DEGs in NDGs incubated with Mock (n3) compared to LDGs incubated with Mock (n3) (Top50).

|  | Gene Symbol | log2 Fold Change | Adjusted *p*-value |
| --- | --- | --- | --- |
| 1 | HBA1 | 9.68147362 | 6.10E-36 |
| 2 | HBB | 9.296379425 | 2.16E-31 |
| 3 | INHBA | -6.165399495 | 2.05E-24 |
| 4 | SLC4A1 | 8.997433236 | 1.25E-22 |
| 5 | HBA2 | 8.974879493 | 1.57E-18 |
| 6 | PAPSS2 | -4.227574875 | 9.11E-17 |
| 7 | SNCA | 5.365932199 | 6.30E-15 |
| 8 | ALAS2 | 8.532070071 | 3.03E-14 |
| 9 | LTF | -6.059319692 | 1.36E-11 |
| 10 | SHANK3 | -4.439764945 | 5.33E-11 |
| 11 | NEURL1 | -4.273263789 | 7.51E-11 |
| 12 | EGR3 | -3.319415674 | 7.51E-11 |
| 13 | HK3 | -2.60948848 | 2.77E-10 |
| 14 | SELENBP1 | 10.01699885 | 7.52E-10 |
| 15 | FOSB | -1.836134525 | 1.87E-09 |
| 16 | CXCL10 | -7.024013482 | 1.87E-09 |
| 17 | NOTCH3 | -4.321763506 | 1.91E-09 |
| 18 | ANK1 | 2.930634355 | 1.19E-08 |
| 19 | BCAT1 | -2.29905463 | 2.26E-08 |
| 20 | EPS8 | -3.87113047 | 2.26E-08 |
| 21 | TRIM58 | 3.638698865 | 2.26E-08 |
| 22 | FZD1 | -4.290104521 | 3.14E-08 |
| 23 | LINC01010 | -4.416235487 | 4.15E-08 |
| 24 | TTYH2 | -3.25699045 | 5.57E-08 |
| 25 | GAS2L3 | -4.391429656 | 6.58E-08 |
| 26 | MMP8 | -5.403035953 | 7.28E-08 |
| 27 | RAB39A | -3.549909822 | 8.27E-08 |
| 28 | LCN2 | -5.368971563 | 1.03E-07 |
| 29 | HSPG2 | -3.200567875 | 1.25E-07 |
| 30 | CSF1R | -3.144744342 | 1.25E-07 |
| 31 | PDZK1IP1 | 4.293965564 | 1.32E-07 |
| 32 | STARD8 | -2.600389619 | 1.47E-07 |
| 33 | STRADB | 3.200591177 | 2.41E-07 |
| 34 | LDLRAD3 | -4.052099297 | 3.13E-07 |
| 35 | EMID1 | -4.909491142 | 3.38E-07 |
| 36 | FAM20A | -4.633231192 | 3.61E-07 |
| 37 | DMTN | 3.549898404 | 3.61E-07 |
| 38 | NPTX2 | -5.778197153 | 3.67E-07 |
| 39 | GCLC | -3.167274714 | 3.96E-07 |
| 40 | FMNL2 | -3.576661988 | 3.96E-07 |
| 41 | NRARP | -2.814001847 | 3.96E-07 |
| 42 | AC104809.2 | -5.385334586 | 3.96E-07 |
| 43 | EGFL7 | -4.392779493 | 4.27E-07 |
| 44 | ALOX15 | 7.336736004 | 4.83E-07 |
| 45 | ATF3 | -3.766542584 | 5.35E-07 |
| 46 | MYCN | -7.687084256 | 5.95E-07 |
| 47 | RUSC2 | -3.132785788 | 6.25E-07 |
| 48 | MELTF | -2.629139807 | 7.21E-07 |
| 49 | DEFA4 | -5.959029613 | 7.80E-07 |
| 50 | PPM1N | -2.777357635 | 8.05E-07 |

Supplemental Table 2. Highly Significant DEGs in NDGs incubated with Mock (n3) compared to LDGs incubated with SARS-CoV-2 (n3) (Top50).

|  | Gene Symbol | log2 Fold Change | Adjusted *p*-value |
| --- | --- | --- | --- |
| 1 | HBB | 8.951500562 | 1.67E-42 |
| 2 | HBA1 | 9.610949373 | 1.62E-31 |
| 3 | HBA2 | 9.402249496 | 7.20E-26 |
| 4 | SLC4A1 | 8.068818176 | 2.98E-21 |
| 5 | INHBA | -5.991564093 | 7.34E-18 |
| 6 | PAPSS2 | -4.241700205 | 6.66E-17 |
| 7 | ALAS2 | 9.1131648 | 6.97E-16 |
| 8 | SHANK3 | -4.488531492 | 6.68E-14 |
| 9 | SNCA | 4.93960969 | 1.19E-13 |
| 10 | LTF | -6.051173735 | 1.41E-11 |
| 11 | BCAT1 | -2.318169856 | 2.21E-11 |
| 12 | FOSB | -1.985846788 | 1.22E-10 |
| 13 | NOTCH3 | -4.461682663 | 2.04E-10 |
| 14 | EGR3 | -3.310904426 | 4.12E-10 |
| 15 | EPS8 | -3.912355578 | 2.41E-09 |
| 16 | NEURL1 | -4.295050677 | 3.51E-09 |
| 17 | DAGLA | -3.21024211 | 5.91E-09 |
| 18 | FZD1 | -4.300995511 | 8.29E-09 |
| 19 | FAM20A | -4.903622419 | 9.12E-09 |
| 20 | HK3 | -2.658422012 | 1.35E-08 |
| 21 | EMID1 | -4.859654047 | 1.70E-08 |
| 22 | TTYH2 | -3.354478308 | 1.99E-08 |
| 23 | SELENBP1 | 8.246728477 | 2.23E-08 |
| 24 | LCN2 | -5.353991966 | 2.23E-08 |
| 25 | PDZK1IP1 | 5.072267636 | 2.23E-08 |
| 26 | TRIM58 | 3.546163697 | 2.23E-08 |
| 27 | HEMGN | 3.605410441 | 3.40E-08 |
| 28 | DMTN | 3.435521349 | 3.40E-08 |
| 29 | GAL3ST4 | 3.35223375 | 5.79E-08 |
| 30 | HBM | 8.591725134 | 6.95E-08 |
| 31 | NRIP3 | -3.516442213 | 7.96E-08 |
| 32 | FMNL2 | -3.501712949 | 8.38E-08 |
| 33 | LINC01010 | -4.388243803 | 9.16E-08 |
| 34 | GAS2L3 | -4.257477488 | 1.12E-07 |
| 35 | MMP8 | -5.463527646 | 1.28E-07 |
| 36 | CSF1R | -3.031514291 | 1.62E-07 |
| 37 | ITGA9 | -3.130564084 | 1.65E-07 |
| 38 | GCLC | -3.239196241 | 2.89E-07 |
| 39 | AC104809.2 | -5.482972821 | 2.89E-07 |
| 40 | HSPG2 | -3.189604183 | 4.09E-07 |
| 41 | STARD8 | -2.631978424 | 4.41E-07 |
| 42 | EGFL7 | -4.319025894 | 4.41E-07 |
| 43 | NMRAL2P | -3.527078517 | 4.67E-07 |
| 44 | NRARP | -2.820719912 | 4.67E-07 |
| 45 | RETN | -4.44528146 | 5.31E-07 |
| 46 | SPINK1 | -5.125769971 | 5.31E-07 |
| 47 | CKB | -2.494689247 | 5.31E-07 |
| 48 | CNTLN | -2.471651441 | 6.74E-07 |
| 49 | ATF3 | -3.724450075 | 6.87E-07 |
| 50 | DEFA4 | -5.925696213 | 7.00E-07 |

Supplemental Table 3. Highly Significant DEGs in NDGs incubated with SARS-CoV-2 (n3) compared to LDGs incubated with Mock (n3) (Top50).

|  | Gene Symbol | log2 Fold Change | Adjusted *p*-value |
| --- | --- | --- | --- |
| 1 | HBA1 | 9.61352157 | 1.69E-31 |
| 2 | HBB | 9.26464228 | 3.79E-27 |
| 3 | HBA2 | 9.2475328 | 2.49E-26 |
| 4 | INHBA | -5.0225978 | 5.36E-22 |
| 5 | SLC4A1 | 8.91800223 | 2.05E-21 |
| 6 | SNCA | 5.26638107 | 1.86E-16 |
| 7 | PAPSS2 | -4.1681024 | 1.31E-15 |
| 8 | ALAS2 | 8.59347561 | 4.82E-14 |
| 9 | SHANK3 | -6.2468202 | 2.34E-13 |
| 10 | NOTCH3 | -3.944659 | 8.80E-13 |
| 11 | LTF | -6.1728609 | 2.34E-12 |
| 12 | VENTX | -2.5766363 | 1.47E-11 |
| 13 | CD300C | -2.6376545 | 1.30E-10 |
| 14 | EPS8 | -4.4657535 | 1.81E-10 |
| 15 | FOSB | -1.8278439 | 2.55E-10 |
| 16 | EGFL7 | -3.5571679 | 2.55E-10 |
| 17 | EGR3 | -3.2153129 | 4.80E-10 |
| 18 | NEURL1 | -3.446288 | 4.84E-10 |
| 19 | SELENBP1 | 9.92387592 | 8.14E-10 |
| 20 | MS4A4A | -4.5600059 | 9.58E-10 |
| 21 | EMID1 | -4.2455982 | 1.04E-09 |
| 22 | NRARP | -3.1772533 | 1.04E-09 |
| 23 | BCAT1 | -2.8223636 | 1.52E-09 |
| 24 | AC090617.5 | -4.6058778 | 1.60E-09 |
| 25 | GAS2L3 | -4.6166887 | 1.64E-09 |
| 26 | SPP1 | -4.5490499 | 2.20E-09 |
| 27 | CXCL10 | -5.9813624 | 2.85E-09 |
| 28 | RAB39A | -3.4835182 | 2.85E-09 |
| 29 | TRIM58 | 3.55646089 | 6.75E-09 |
| 30 | HK3 | -2.472485 | 8.91E-09 |
| 31 | MYCN | -3.9556676 | 1.52E-08 |
| 32 | LYPD2 | -5.867223 | 1.60E-08 |
| 33 | P2RX7 | -3.3007317 | 1.81E-08 |
| 34 | LINC00346 | -6.032327 | 1.81E-08 |
| 35 | EPB42 | 9.05533924 | 2.36E-08 |
| 36 | ANK1 | 2.74396041 | 4.60E-08 |
| 37 | L1TD1 | -5.1416206 | 5.42E-08 |
| 38 | CSF1R | -3.1364102 | 6.62E-08 |
| 39 | LCN2 | -5.2713137 | 9.73E-08 |
| 40 | DMTN | 3.42859059 | 9.74E-08 |
| 41 | LPL | -6.2076215 | 1.00E-07 |
| 42 | RBKS | -1.6926768 | 1.45E-07 |
| 43 | CEACAM6 | -5.2148275 | 1.48E-07 |
| 44 | NPTX2 | -5.2240585 | 1.48E-07 |
| 45 | MIR3945HG | -2.9703302 | 1.51E-07 |
| 46 | STARD8 | -2.3651775 | 1.55E-07 |
| 47 | PTX4 | -5.4507102 | 1.95E-07 |
| 48 | MMP8 | -5.0844527 | 2.04E-07 |
| 49 | LGALS12 | 3.49656648 | 2.15E-07 |
| 50 | LINC01010 | -4.797043 | 3.02E-07 |

Supplemental Table 4. Highly Significant DEGs in NDGs incubated with SARS-CoV-2 (n3) compared to LDGs incubated with SARS-CoV-2 (n3) (Top50).

|  | Gene Symbol | log2 Fold Change | Adjusted *p*-value |
| --- | --- | --- | --- |
| 1 | HBA2 | 9.67712183 | 9.09E-41 |
| 2 | HBB | 8.924876805 | 3.93E-35 |
| 3 | HBA1 | 9.547838625 | 1.60E-27 |
| 4 | SLC4A1 | 7.993751243 | 1.18E-19 |
| 5 | SHANK3 | -6.281973789 | 7.51E-16 |
| 6 | ALAS2 | 9.181068822 | 1.05E-15 |
| 7 | PAPSS2 | -4.17864903 | 1.05E-15 |
| 8 | SNCA | 4.844095463 | 2.88E-15 |
| 9 | NOTCH3 | -4.080258411 | 1.04E-14 |
| 10 | INHBA | -4.831270439 | 1.04E-14 |
| 11 | EMID1 | -4.204839333 | 1.47E-12 |
| 12 | LTF | -6.16090327 | 3.07E-12 |
| 13 | FOSB | -1.973971013 | 8.99E-12 |
| 14 | EPS8 | -4.503064702 | 1.01E-11 |
| 15 | BCAT1 | -2.837600173 | 1.19E-11 |
| 16 | EGFL7 | -3.48053178 | 8.62E-11 |
| 17 | SPP1 | -4.391555649 | 1.10E-10 |
| 18 | CD300C | -2.639330437 | 1.59E-10 |
| 19 | VENTX | -2.525698652 | 8.16E-10 |
| 20 | NRARP | -3.18104129 | 1.56E-09 |
| 21 | EGR3 | -3.203385778 | 2.42E-09 |
| 22 | HEMGN | 3.52037556 | 2.73E-09 |
| 23 | DMTN | 3.317482427 | 2.73E-09 |
| 24 | L1TD1 | -5.363001155 | 3.60E-09 |
| 25 | GAS2L3 | -4.479658161 | 3.96E-09 |
| 26 | TRIM58 | 3.468039314 | 7.71E-09 |
| 27 | MS4A4A | -4.54676793 | 8.04E-09 |
| 28 | AC090617.5 | -4.518891046 | 8.04E-09 |
| 29 | DAGLA | -3.390930549 | 8.91E-09 |
| 30 | LINC00346 | -6.085464839 | 1.47E-08 |
| 31 | EPB42 | 9.134748177 | 1.89E-08 |
| 32 | LCN2 | -5.253750655 | 2.48E-08 |
| 33 | SELENBP1 | 8.157402876 | 2.54E-08 |
| 34 | LPL | -6.254074382 | 2.83E-08 |
| 35 | PPP1R17 | -4.451258812 | 2.86E-08 |
| 36 | GAL3ST4 | 3.499499007 | 2.89E-08 |
| 37 | HBM | 8.746625774 | 3.66E-08 |
| 38 | SPINK1 | -5.037520441 | 3.81E-08 |
| 39 | NEURL1 | -3.465573465 | 3.82E-08 |
| 40 | RBBP8 | -1.856488509 | 4.77E-08 |
| 41 | P2RX7 | -3.213364773 | 5.55E-08 |
| 42 | GLA | -2.077429315 | 8.19E-08 |
| 43 | CSF1R | -3.020263596 | 8.46E-08 |
| 44 | FAM20A | -4.04634415 | 1.18E-07 |
| 45 | AC090617.3 | -3.898456828 | 1.59E-07 |
| 46 | HPGDS | -5.330088044 | 1.63E-07 |
| 47 | CEACAM6 | -5.08737656 | 1.70E-07 |
| 48 | HK3 | -2.517555529 | 1.72E-07 |
| 49 | PDZK1IP1 | 4.819491752 | 1.98E-07 |
| 50 | LYPD2 | -6.128116494 | 2.21E-07 |
